# Evidence-Based Topical Therapy for Facial Scars in Diverse Skin Types

**DOI:** 10.7759/cureus.86343

**Published:** 2025-06-19

**Authors:** Sarah Kazemeini, Ahmed Nadeem-Tariq, Anahat Luthra, Raisa Suha, Arun Turna, Jennifer Easterly, Sahara Pokharel, Sophia Muqaddas, Monia Kazemeini

**Affiliations:** 1 Medical Education, Kirk Kerkorian School of Medicine, University of Nevada, Las Vegas, Las Vegas, USA; 2 Otolaryngology - Head and Neck Surgery, Kirk Kerkorian School of Medicine, University of Nevada, Las Vegas, Las Vegas, USA; 3 Medicine, Nova Southeastern University Dr. Kiran C. Patel College of Osteopathic Medicine, Tampa, USA; 4 Medicine, Stony Brook University, Stony Brook, USA; 5 Medicine, California Health Sciences University College of Osteopathic Medicine, Clovis, USA; 6 Medicine, Medical College of Georgia, Augusta University, Augusta, USA; 7 Medicine, Lincoln Memorial University - DeBusk College of Osteopathic Medicine, Knoxville, USA; 8 Mechanical Engineering, University of Nevada, Las Vegas, Las Vegas, USA

**Keywords:** corticosteroids, facial scars, hypertrophic scars, keloids, retinoids, scar management, silicone gel, skin of color, topical therapy

## Abstract

Facial trauma repair frequently results in scarring that can significantly impact psychological well-being, particularly in highly visible areas, such as the face. While topical agents provide a non-invasive means of optimizing scar outcomes, clinical guidance regarding their use remains limited, especially for diverse skin types. This narrative review synthesizes findings from PubMed, Google Scholar, and Embase, prioritizing randomized controlled trials, meta-analyses, and expert consensus. Evidence supports the use of silicone gel, corticosteroids, retinoids, vitamin C, niacinamide, and onion extract for scar modulation. A timeline-based model was developed to guide agent selection across healing phases, emphasizing early intervention after re-epithelialization and tailored regimens for patients with Fitzpatrick skin types IV-VI. This review proposes a phase-specific approach to facial scar optimization that integrates skin phototype considerations. Further research is needed to validate the safety and efficacy of combinatory topical regimens across diverse populations.

## Introduction and background

Facial trauma repair typically results in visible scarring that can significantly impact a patient’s psychological well-being, social functioning, and quality of life. Because the face is both highly visible and functionally dynamic, even subtle postoperative changes can be perceived as disfiguring. Studies have shown that facial scars may contribute to diminished self-esteem, social withdrawal, and even clinical depression [1-5]. These effects are compounded by the complexity of facial anatomy, regional variation in skin thickness, and the tension placed on healing wounds during everyday facial movements [6,7].

Patients with skin of color face additional challenges, including a higher risk of post-inflammatory hyperpigmentation (PIH), hypertrophic scarring, and keloid formation. PIH refers to darkened areas of skin following inflammation or injury, while hypertrophic scars and keloids involve excessive collagen deposition that leads to raised, firm scar tissue. These complications are more prevalent in individuals with Fitzpatrick skin types IV-VI and can significantly affect long-term aesthetic outcomes [8-10]. Despite this, most scar management protocols are not tailored to individual patient characteristics or skin types, and evidence-based approaches to topical scar optimization remain underutilized in standard postoperative care. Moreover, there is a critical need for further research to evaluate the safety and efficacy of combination topical regimens, particularly in populations with skin of color, who remain underrepresented in clinical trials.

Topical agents represent a low-risk, accessible intervention that can be integrated into postoperative regimens to improve scar quality. Despite the widespread use of topical therapies, current protocols often fail to align with the biologic stages of wound healing and ignore skin phototype-specific factors. This review introduces a structured, timeline-based approach that considers both the healing phase and melanin biology. It addresses critical practice gaps by providing stratified recommendations based on risk of hypertrophic scarring, Fitzpatrick type, and evidence of agent synergy. Few existing protocols provide a framework that guides layering strategies or distinguishes between agents suitable for early versus late-phase use in patients with darker skin tones.

To address this gap, a narrative review of the most commonly used topical therapies for scar management following maxillofacial fracture repair was conducted. Sources were identified through comprehensive searches of PubMed, Google Scholar, and Embase using terms such as “facial scar,” “postoperative scar management,” “topical agents,” “skin of color,” and “post-inflammatory hyperpigmentation.” We prioritized randomized controlled trials, meta-analyses, and expert consensus statements. Articles were included based on clinical relevance to facial scar management, and emphasis was placed on studies evaluating agent efficacy across different Fitzpatrick skin types. The search was limited to English-language studies published through May 2025.

## Review

Overview of scar formation in facial surgery

Cutaneous wound healing proceeds through four sequential phases: hemostasis, inflammation, proliferation, and remodeling [11]. Hemostasis begins immediately after injury to prevent blood loss and initiate tissue stabilization. Inflammation follows, recruiting immune cells and releasing cytokines that prime the wound bed for repair [12,13].

During the proliferative phase, fibroblasts generate extracellular matrix (ECM) components, notably collagen, which support new tissue growth and angiogenesis. This is a critical window for therapeutic intervention, as excessive fibroblast activity can predispose to hypertrophic scars. In the remodeling phase - lasting weeks to months - type III collagen is replaced by stronger type I collagen, and the ECM is reorganized to improve tensile strength [14].

The facial region has features that generally support efficient healing, including robust vascular supply and high sebaceous gland density [15,16]. However, certain patient- and procedure-specific factors can increase the likelihood of adverse scarring. High-tension wound closures, for instance, elevate the risk of fibroproliferative responses. In patients with darker skin tones, this risk is compounded by biological tendencies toward increased melanocyte activity and altered vitamin D metabolism, which may influence both pigmentary changes and fibroblast behavior [17,18]. Therefore, even in a region with strong regenerative potential, thoughtful attention to wound mechanics and skin-specific healing patterns is essential to optimize postoperative outcomes, especially among patients with skin of color.

Silicone gel and silicone sheets

Silicone gel and silicone sheets exert their beneficial effects on scar tissue through a combination of occlusion, hydration, and collagen modulation. Occlusion helps maintain stratum corneum hydration, reducing transepidermal water loss and creating an environment that regulates fibroblast activity and collagen deposition. This process also suppresses mast cell activity, which may contribute to a reduction in erythema and pruritus [19,20]. Studies have shown that consistent use of silicone gel sheeting can lead to flatter, less erythematous, and more pliable scars [21-23]. Given its non-invasive nature, ease of use, and minimal side effect profile, silicone remains a first-line option for scar management in both early and mature hypertrophic or keloid scars [23].

Topical corticosteroids

Topical corticosteroids reduce scar formation by inhibiting fibroblast proliferation, inducing fibroblast apoptosis, and suppressing inflammation through the inhibition of transcription factors such as nuclear factor kappa-light-chain-enhancer of activated B cells (NF-κB) and activator protein 1 (AP-1) [24]. They also downregulate angiogenic signaling by suppressing vascular endothelial growth factor (VEGF) and modulate ECM turnover via transforming growth factor-beta 1 (TGF-β1) and basic fibroblast growth factor (bFGF) regulation [24]. These mechanisms support their role in managing early hypertrophic scars, where they are often used to treat raised or itchy lesions [25]. Their anti-inflammatory properties are key in preventing excessive collagen deposition and tissue remodeling that can lead to hypertrophic changes [26,27]. Furthermore, corticosteroids offer symptomatic relief from pruritus, improving patient comfort during early healing stages [26].

While corticosteroids are effective, their use must be managed carefully to avoid potential adverse effects, particularly in facial scars. Prolonged use, especially on sensitive facial skin, can lead to side effects such as skin thinning, which may worsen the appearance of scars [27]. To mitigate these risks, it is often recommended that lower-potency corticosteroids be used and treatment duration be limited [27]. Regular monitoring is essential to balance the benefits of scar reduction with the risks associated with long-term use. Topical corticosteroids are an important treatment option for raised and itchy scars, especially when applied during the early stages of scar formation.

Retinoids

Retinoids, derivatives of vitamin A, are widely used topical agents in scar remodeling due to their potent effects on epidermal turnover, texture improvement, and attenuation of PIH. Topical retinoids act by binding to epidermal keratinocytes and retinoid X receptors. Preclinical studies have shown that they modulate the transcription of several genes involved in cellular proliferation, differentiation, and ECM remodeling [28]. Specifically, in vitro evidence suggests that retinoids accelerate epidermal turnover by promoting proliferation and differentiation of keratinocytes and suppressing melanin transport to epidermal cells [28]. This assists in eliminating patches of hyperpigmentation [29,30].

Clinically, topical retinoid treatment in frequency and timing must be carefully thought through due to the high irritative potential. Topical tretinoin treatment is most often associated with side effects of erythema, peeling, dryness, burning, stinging, and increased photosensitivity [29]. These reactions predominantly occur within the first few weeks of therapy, with maximal irritation typically peaking around two weeks after initiation [31]. To improve tolerability, the authors recommend strategies such as initiating therapy on lower strengths (e.g., 0.025% or 0.05% tretinoin) and retinoid application less often [29]. A gradual increase of dose and frequency, coupled with supportive skincare and adequate patient education, is crucial to maintain compliance long enough for topical retinoids to exert their full therapeutic benefit.

Onion extract

Onion extract provides a mild anti-inflammatory effect primarily through the bioactivity of its flavonoids, particularly quercetin and kaempferol. Preclinical studies suggest these flavonoids may modulate fibroblast proliferation and collagen synthesis by suppressing TGF-β (TGF-β1 and TGF-β2) and insulin-like growth factor-1 (IGF-1) signaling pathways [32]. Given the central role of TGF-β and IGF-1 in promoting excessive collagen deposition, inhibiting these pathways could result in reduced scar thickness and improved texture. Additional in vitro research has shown that flavonoids can inhibit histamine release from basophils, which could reduce local inflammation and fibroblast activation [33]. Bringing these cellular mechanisms to translation, one can hypothesize that onion extract is particularly valuable during the active stage of remodeling in wound healing, with the possibility of stabilizing, or even reversing, initial pathological scar formation before ECM deposition has become too extensive.

The clinical evidence regarding the effectiveness of onion extract remains inconsistent. Some studies have suggested that onion extract may modestly improve scar texture and reduce hypertrophic scarring, though many of these investigations suffer from methodological limitations, such as small sample sizes and lack of controls [33]. In addition, although onion extract is generally safe and tolerable in a majority of instances, the potential for slight skin irritation indicates a practical limitation toward broader application [34]. In light of these factors, onion extract-containing preparations likely have their greatest utility in the form of supplementary or adjunctive therapies, particularly in low-risk, cosmetically sensitive areas, where minimal improvements in scar quality can have a meaningful impact on patient satisfaction.

Vitamin C

Vitamin C plays an important role in collagen synthesis and protection against oxidative stress. As a required cofactor for prolyl and lysyl hydroxylase enzymes, vitamin C is essential for the post-translational hydroxylation of collagen molecules, a step needed to stabilize the collagen triple-helix and promote proper fiber cross-linking [35]. In wounds and scars, adequate availability of vitamin C allows fibroblasts to produce more stable, highly ordered bundles of collagen. However, a deficiency in vitamin C yields less-than-optimally hydroxylated collagen with low tensile strength [35]. Topical application of vitamin C may increase local skin concentrations, potentially supporting collagen synthesis and improving scar quality. In addition, vitamin C is a potent antioxidant that scavenges reactive oxygen species [35]. Topical application may improve local skin concentrations, though clinical evidence for scar remodeling remains limited and somewhat variable.

Following trauma, the wound site experiences oxidative stress from inflammation and ultraviolet (UV) exposure, which can damage cells and exacerbate scar hyperpigmentation. Vitamin C helps neutralize free radicals and regenerates other antioxidants, like vitamin E, thereby mitigating oxidative damage in healing skin [36]. This antioxidant property is thought to reduce post-inflammatory melanogenesis and excessive fibroplasia. In vitro studies also suggest that vitamin C reduces post-inflammatory melanogenesis by inhibiting tyrosinase and reducing oxidized melanin intermediates [35,37]. 

A crucial consideration with topical vitamin C is the formulation, as not all vitamin C products are created equal in stability or bioavailability. L-ascorbic acid, the active form of vitamin C, is a highly unstable molecule that can oxidize and degrade easily when exposed to air, light, or higher pH environments [35]. Effective topical formulations must, therefore, stabilize ascorbic acid to preserve its potency until it penetrates the skin [38]. Many over-the-counter “vitamin C” creams use derivatives such as magnesium ascorbyl phosphate, ascorbyl glucoside, or tetrahexyldecyl ascorbate, which are more stable but must be enzymatically converted to active ascorbic acid within the skin [36]. Overall, the formulation and stability of topical vitamin C are key determinants of its success in scar therapy. Therefore, clinicians selecting vitamin C for facial scars should use evidence-based formulations and educate patients that consistent application over several months is needed to see the full spectrum of scar improvements.

Niacinamide

Niacinamide (vitamin B3) and azelaic acid are two well-tolerated topical agents that serve complementary roles in scar care, particularly for addressing inflammation and dyschromia in healing facial scars. Niacinamide is a multifaceted ingredient with anti-inflammatory, antioxidant, and pigment-regulating properties. In the skin, niacinamide reduces the production of inflammatory mediators (like IL-6) and helps restore the skin barrier, thereby calming redness and irritation in the scar site [39]. It also has a direct effect on pigmentation, as niacinamide inhibits the transfer of melanin from melanocytes to surrounding keratinocytes in the epidermis [40]. By interfering with this melanosome transfer, niacinamide prevents the diffusion of pigment into scar tissue, helping to prevent and lighten PIH. These mechanisms make niacinamide especially valuable for patients with skin of color, who often develop dark marks in scars (PIH), and who may benefit from an anti-inflammatory approach to scar prevention. 

Several small-scale randomized controlled trials and observational studies support niacinamide’s pigment-fading and anti-inflammatory effects in human skin. Histologic analyses in these trials confirmed that niacinamide-treated skin had fewer melanin granules and less inflammatory cell infiltrate than controls [41,42]. Translating these findings to scars: applying niacinamide on a healing facial scar can similarly reduce the likelihood of persistent brown discoloration and can soothe the underlying inflammation that drives both hyperpigmentation and hypertrophic changes. Moreover, niacinamide can help improve the color uniformity of scars over time, a key cosmetic goal, by gradually evening out dark spots or redness. Given its broad benefits and excellent safety, niacinamide has become a staple ingredient in scar creams and serums aimed at reducing PIH and improving overall scar appearance.

A major advantage of niacinamide is its long-term tolerability and safety on the face, which makes it ideal for prolonged use during the scar remodeling period [43]. Niacinamide is a form of vitamin B3 and is non-irritating. Even at 5% concentrations in trials, it produced no notable side effects apart from occasional mild stinging, which was comparable to vehicle controls [44-46]. This very low irritation profile supports daily use without risk and enables clinicians to use niacinamide reliably as part of other scar treatments without worsening inflammation [47-50].

A timeline-based framework for agent selection can guide personalized scar therapy across healing stages. Table 1 presents agent recommendations, derived from a combination of randomized trials, expert consensus statements, and author synthesis to support personalized scar management.

**Table 1:** Timeline-Based Recommendations for Topical Agent Selection Based on Scar Properties and Risk Factors Data adapted from Puri and Talwar [19], Lin and Lai [24], Zasada and Budzisz [28], Pullar et al. [35], Marques et al. [39], and Bronte et al. [50]. Some entries were directly extracted from study findings (e.g., randomized controlled trials or expert consensus), while others were modified based on the author’s interpretation to support clinical applicability; no direct reproduction.

Timeline	Scar Properties	Risk Factors	Recommended Agents
Immediate (0-4 weeks)	All	All Skin Types	Silicone sheeting [19], Silicone gel [19], Broad-Spectrum Sunscreen [50]
		Fitzpatrick IV-VI	Vitamin C [35], Niacinamide [39]
		History of Keloids or Hypertrophic Scars	Early Monitoring [50]
Early (4-12 weeks)	Hyperpigmented Scars	Fitzpatrick IV-VI	Vitamin C [35], Niacinamide [39], Azelaic Acid [50]
	Erythematous or Hypertrophic Scars	History of Keloids or Hypertrophic Scars	Corticosteroids [24]
Remodeling Phase (3-12 months)	Matured or Improved Scars	All Skin Types	Retinoids [28], Vitamin C [35,39]
	Non-improving Hyperpigmented or Hypertrophic Scars	Fitzpatrick IV-VI and History of Keloids or Hypertrophic Scars	Dermatology Referral [50]

Table 2 integrates published data and the author's interpretation to summarize key characteristics of topical agents used in facial scar care.

**Table 2:** Summary of Topical Agents for Scar Optimization Data adapted from Puri and Talwar [19], Lin and Lai [24], Zasada and Budzisz [28], Pullar et al. [35], Marques et al. [39], and Bronte et al. [50]. Some entries were directly extracted from study findings (e.g., randomized controlled trials or expert consensus), while others were modified based on the author’s interpretation to enhance clinical relevance; no direct reproduction.

Topical Agent	Mechanism of Action	Best Timing of Use	Considerations in Skin of Color
Silicone Gel/Sheets [19]	Occlusion, hydration of stratum corneum, fibroblast modulation	Early remodeling phase (post-epithelialization)	Well-tolerated across skin types
Topical Corticosteroids [24]	Inhibits fibroblast proliferation, reduces collagen synthesis, anti-inflammatory	Early hypertrophic stage	Use low-potency formulations to reduce the risk of hypopigmentation
Retinoids [28]	Increases epidermal turnover, stimulates collagen, reduces post-inflammatory hyperpigmentation (PIH)	Post-inflammatory phase; avoid during the open wound phase	May cause irritation; start with less potent derivatives
Onion Extract [32]	Anti-inflammatory, inhibits fibroblast proliferation, reduces scar height	Early scar maturation phase	Generally safe, low risk of PIH
Vitamin C [35]	Antioxidant, collagen synthesis, reduces hyperpigmentation	Post-epithelialization, for pigmentation and texture refinement	Can be combined with other agents for PIH
Niacinamide [39]	Inhibits melanosome transfer, increases ceramide synthesis, anti-inflammatory	Post-epithelialization, during early pigmentation changes	Safe for long-term use; well-studied in diverse skin tones

Special considerations in skin of color

Individuals with skin of color may face a higher risk of adverse scarring outcomes due to both biological factors and limited representation in clinical research. Melanin, the pigment primarily responsible for skin color, is more densely distributed and stored in larger, dispersed melanosomes in individuals with Fitzpatrick skin types IV-VI [45,46]. While melanin provides photoprotection by absorbing UV radiation, it also plays a key role in post-inflammatory pigmentary changes. The Fitzpatrick Skin Phototype Scale, which categorizes skin based on its response to UV exposure, offers a framework for assessing photodamage risk and guiding dermatologic care [47].

Increased melanin reactivity in the skin of color predisposes patients to PIH following trauma or inflammation. This manifests as irregular melanin deposition and often presents as persistent hyperpigmented patches, particularly in the context of acne or surgical injury [48]. PIH can be cosmetically distressing, and is often more prominent, widespread, and resistant to treatment in darker skin types. Therefore, early and sustained inflammation control is critical to minimizing pigmentary sequelae.

Beyond pigmentary concerns, patients with skin of color are also more likely to develop fibroproliferative scarring. Keloids arise from dysregulated collagen production and fibroblast proliferation [49]. First-line treatments often include intralesional corticosteroids, which reduce inflammation and suppress fibroblast activity. However, these therapies may be limited by side effects in patients with Fitzpatrick IV-VI skin, including hypopigmentation, dermal atrophy, and epidermal thinning [50].

Management of PIH and fibroproliferative scars in skin of color requires an individualized approach. For pigmentary changes, treatment begins with addressing the underlying inflammatory trigger. Topical therapies such as hydroquinone, azelaic acid, retinoids, kojic acid, and licorice extract can be effective, especially when combined with strict photoprotection. More resistant pigmentation may require procedural interventions, including chemical peels and laser therapies [51]. Clinicians must remain vigilant for potential adverse effects such as contact dermatitis or nail discoloration [51].

Establishing clear, culturally sensitive communication is essential when treating patients with skin of color. Providing realistic timelines and discussing potential side effects builds trust and improves adherence. A patient-centered approach that acknowledges the unique healing patterns of skin of color enhances both outcomes and patient satisfaction.

Timing, layering, and adherence strategies: a proposed protocol

The timing of scar therapy initiation is a crucial determinant of cosmetic outcomes following facial trauma repair. Current clinical guidance supports introducing topical agents once re-epithelialization is complete, which is typically between 10 and 14 days postoperatively. This avoids disruption of the newly formed epidermal barrier [52,53]. Initiating treatment too soon may interfere with wound closure, while excessive delay may reduce the efficacy of interventions. Among available treatments, silicone gel sheeting remains the cornerstone of hypertrophic scar and keloid management when applied during this important stage of healing.

Duration and consistency of therapy are equally important. Extended application of silicone-based products has been shown to significantly reduce scar erythema and thickness, while improving overall texture and color, particularly in highly visible regions such as the face [54]. Evidence also supports the use of layering strategies, where multiple agents are employed in a phased and complementary manner. For example, silicone gel applied during the day helps maintain hydration and barrier function, creating an optimal microenvironment for scar remodeling. At night, topical retinoids such as tretinoin may be introduced to enhance collagen turnover and reduce pigmentary abnormalities. Concurrent use of antioxidants like vitamin C serum beneath silicone during daytime hours can help reduce oxidative stress and suppress melanogenesis [52]. While promising, these approaches require further research to confirm long-term efficacy and safety. Direct comparative studies assessing the synergistic or additive effects of layering agents - such as silicone, retinoids, and vitamin C - are currently limited. Most available evidence evaluates these agents individually, and future research should prioritize understanding their combined use in scar management.

Non-invasive procedural therapies can also increase the benefits of topicals. Intralesional triamcinolone remains a widely used first-line agent for hypertrophic scars due to its ability to inhibit fibroblast proliferation and accelerate collagen degradation [55]. Furthermore, adjunctive use of topical retinoids, such as all-trans-retinoic acid, can mitigate steroid-induced dermal atrophy and support ongoing matrix remodeling [56-58]. When integrated thoughtfully, topical therapies not only improve scar outcomes independently but also potentiate the effects of procedural interventions, offering a comprehensive approach to aesthetic recovery.

## Conclusions

Postoperative scar management following facial fracture repair persists as a changing domain of care. This narrative review emphasizes the use of topical agents, ranging from silicone gel to corticosteroids, as low-risk interventions when used in a phase-specific timeline of application. Patients with skin of color present unique challenges that are often overlooked in current scar management protocols. By utilizing considerations for melanin biology and steroid sensitivity, clinicians can offer more effective treatment for all patient populations.

The timeline-based model proposed in this review not only systematizes the initiation and layering of topical agents, but also reveals critical gaps in current clinical practice. Despite widespread use of agents like silicone gel and corticosteroids, protocols rarely integrate wound healing biology with skin phototype-specific considerations. For example, recommendations for retinoid or antioxidant use often fail to account for the heightened risk of irritation or pigment alteration in Fitzpatrick IV-VI populations. Similarly, while intralesional corticosteroids are commonly used, their long-term impact when paired with topical agents in skin of color remains poorly studied. This model emphasizes the need for prospective studies that assess therapeutic efficacy stratified by skin type, scar morphology, and intervention timing. While this review proposes a generalizable framework for topical scar therapy, clinicians should adapt these recommendations based on institutional resources, patient demographics, and local practice patterns to ensure optimal outcomes in their specific care settings.
